# Hypernatremia in Hyperglycemia: Clinical Features and Relationship to Fractional Changes in Body Water and Monovalent Cations during Its Development

**DOI:** 10.3390/jcm13071957

**Published:** 2024-03-28

**Authors:** Brent Wagner, Todd S. Ing, Maria-Eleni Roumelioti, Ramin Sam, Christos P. Argyropoulos, Susie Q. Lew, Mark L. Unruh, Richard I. Dorin, James H. Degnan, Antonios H. Tzamaloukas

**Affiliations:** 1 Division of Nephrology, Department of Medicine, University of New Mexico School of Medicine, Albuquerque, NM 87122, USA; brwagner@salud.unm.edu (B.W.); mroumelioti@salud.unm.edu (M.-E.R.); cargyropoulos@salud.unm.edu (C.P.A.); 2 Kidney Institute of New Mexico, University of New Mexico Health Sciences Center, Albuquerque, NM 87122, USA; 3 Raymond G. Murphy Veterans Affairs Medical Center, Albuquerque, NM 87108, USA; 4 Department of Medicine, Stritch School of Medicine, Loyola University Chicago, Maywood, IL 60153, USA; 5 Department of Medicine, Zuckerberg San Francisco General Hospital, University of California in San Francisco School of Medicine, San Francisco, CA 94110, USA; ramin.sam@ucsf.edu; 6 Department of Medicine, School of Medicine and Health Sciences, George Washington University, Washington, DC 20037, USA; sqlew@gwu.edu; 7 Department of Medicine, University of New Mexico School of Medicine, Albuquerque, NM 87122, USA; mlunrugh@salud.unm.edu; 8 Department of Medicine, Division of Endocrinology, Raymond G. Murphy Veterans Affairs Medical Center, University of New Mexico, Albuquerque, NM 87108, USA; rdorin@salud.unm.edu; 9 Department of Mathematics and Statistics, University of New Mexico, Albuquerque, NM 87131, USA; jamdeg@unm.edu; 10 Research Service, Department of Medicine, Raymond G. Murphy Veterans Affairs Medical Center, University of New Mexico School of Medicine, Albuquerque, NM 87108, USA

**Keywords:** hyperglycemia, hypernatremia, osmotic diuresis, sodium in fluids lost, potassium in fluids lost

## Abstract

In hyperglycemia, the serum sodium concentration (*[Na]_S_*) receives influences from (a) the fluid exit from the intracellular compartment and thirst, which cause *[Na]_S_* decreases; (b) osmotic diuresis with sums of the urinary sodium plus potassium concentration lower than the baseline euglycemic *[Na]_S_*, which results in a *[Na]_S_* increase; and (c), in some cases, gains or losses of fluid, sodium, and potassium through the gastrointestinal tract, the respiratory tract, and the skin. Hyperglycemic patients with hypernatremia have large deficits of body water and usually hypovolemia and develop severe clinical manifestations and significant mortality. To assist with the correction of both the severe dehydration and the hypovolemia, we developed formulas computing the fractional losses of the body water and monovalent cations in hyperglycemia. The formulas estimate varying losses between patients with the same serum glucose concentration (*[Glu]_S_*) and *[Na]_S_* but with different sums of monovalent cation concentrations in the lost fluids. Among subjects with the same *[Glu]_S_* and *[Na]_S_*, those with higher monovalent cation concentrations in the fluids lost have higher fractional losses of body water. The sum of the monovalent cation concentrations in the lost fluids should be considered when computing the volume and composition of the fluid replacement for hyperglycemic syndromes.

## 1. Introduction

In hyperglycemic syndromes, the serum sodium concentration (*[Na]_S_*) may receive influences from pathophysiologic processes directly and indirectly linked to hyperglycemia. The following three processes are directly related to hyperglycemia: (a) The osmotic fluid flux from the intracellular into the extracellular compartment, secondary to the extracellular solute (glucose) gain. This process, which causes a decrease in the *[Na]_S_* [1,2,3], is present in all hyperglycemic episodes but may be masked by other influences in some episodes. (b) The loss of water, sodium, and potassium through glucosuric osmotic diuresis causing a *[Na]_S_* increase [4,5]. This process operates in hyperglycemic episodes occurring in patients with preserved renal function. (c) Thirst from hypertonicity and hypovolemia leading to fluid intake. The effects of this process, which causes a *[Na]_S_* decrease, are usually less evident than the other two effects [6]. Processes affecting the *{Na]_S_* operating in some, but not all, hyperglycemic episodes include the intake of glucose, electrolytes, and fluids and the losses through the gastrointestinal tract, the respiratory tract, and the skin. 

At presentation with hyperglycemia, the *[Na]_S_* has usually received influences from more than one of the pathophysiological processes listed above. Computing the effect of the hypertonic hyponatremia caused by an extracellular glucose gain on the *[Na]_S_* is important because this effect is reversed by the correction of the hyperglycemia. Oligo-anuric patients have minimal or no losses of water and electrolytes during the development of hyperglycemia. The reversal of hypertonic hyponatremia was demonstrated in this set of patients by the correction of the hyperglycemia with insulin, no other therapeutic measures taken, and maintenance of the same body weight before and after treatment [7]. The correction of the changes in the *[Na]_S_* caused by other pathophysiologic processes requires additional measures. 

In patients with preserved renal function, the replacement of water, sodium and potassium losses, and the normalization of the *[Na]_S_* constitute critical parts of the treatment, along with the correction of the hyperglycemia and of the associated acid–base disorders [8,9,10,11,12,13,14,15,16,17]. In these patients, water and electrolytes are lost through osmotic diuresis. In osmotic diuresis, including specifically hyperglycemic diuresis [18,19,20], the sum of the concentrations in the urine of sodium (*[Na]_U_*) plus potassium (*[K]_U_*) is lower than the baseline euglycemic *[Na]_S_* [4,5] resulting in a rise in the *[Na]_S_*. Note: In osmotic diuresis, urine osmolality is routinely higher than serum osmolality [5]. Consequently, the use of osmolality-based solute and water clearance values to compute the effect of osmotic diuresis on the *[Na]_S_* leads to erroneous conclusions; the proper biochemical parameter in this case is the sum *[Na]_U_* + *[K]_U_* [21]. When computing the volume and composition of replacement solutions in hyperglycemic syndromes, the volume of osmotic diuresis and its monovalent cation concentrations merit consideration. 

This review focuses on hypernatremia in presentation with hyperglycemia. In such cases, hypernatremia usually develops when the effect of osmotic diuresis remains the dominant influence on the *[Na]_S_* and indicates a profound body water deficit. Other mechanisms may also contribute to the development of hypernatremia and will be presented later in this article. Our report has two aims: (a) to investigate the predisposing conditions, clinical features, and outcomes of hyperglycemic syndromes presenting with hypernatremia by reviewing published patient series and case reports, and (b) to present a method for calculating the fractional losses, usually in the urine, of water, sodium, and potassium, which result in any combination of the hyperglycemic serum glucose concentration (*[Glu]_S_*) and *[Na]_S_*.

## 2. Reports of Hypernatremia in Hyperglycemia

We searched for published studies and case reports on hyperglycemia with a measured *[Na]_S_* in the range of hypernatremia in diabetic ketoacidosis (DKA), nonketotic hyperosmolar state (HHS), and combined DKA-HHS, which in one study was detected in 325 of 1211 (27%) patients hospitalized for hyperglycemia and was associated with higher in-hospital mortality than either DKA or HHS [22]. Our search included PubMed reports under the term “hypernatremia in hyperglycemia” and the lists of references of the PubMed reports. We included in this report the following: (a) studies of hyperglycemic syndromes with a mean *[Na]_S_* ≥ 145 mmol/L; (b) studies of hyperglycemic syndromes analyzing hypernatremia as a contributor to specific pathophysiologic entities; and (c) case reports of hyperglycemia with a *[Na]_S_* ≥ 145 mmol/L. 

The serum tonicity (effective osmolarity) was calculated using the formula 2 × *[Na]_S_* + *[Glu]_S_* expressed in mmol/L [23]. The value of the *[Na]_S_* that would be obtained by the correction of hyperglycemia to a *[Glu]_S_* of 5.6 mmol/L (100 mg/dL) without any changes in the body water, sodium, and potassium (*[Na]_Cor_*) was calculated by the formula of Al-Kudsi and co-investigators [24], as will be explained later in this report. The statistical analysis of the case reports included testing whether their distribution was normal or not by the Shapiro–Wilk test [25] and the computation of the appropriate distribution parameters. 

## 3. Reports of Hyperglycemic Syndromes with Mean *[Na]_S_* in the Hypernatremic Range

The mean *[Na]_S_* was ≥145 mmol/L in three reports on HHS [26,27,28]. Table 1 shows the mean values of the *[Glu]_S_*, *[Na]_S_*, serum tonicity, and *[Na]_Cor_* of these studies. 

The review of Danowski and Nabarro [26] contained 21 cases and the study of Halmos and co-investigators [27] had 6 cases of hyperglycemia and hypernatremia. The biochemical values and clinical status of each case in the two publications were analyzed in the case reports section.

Piniés and co-authors [28] analyzed the precipitating conditions, clinical picture, and outcomes of 132 patients with HHS. A previous history of diabetes was found in 51% of the patients. A “precipitating factor” for HHS was identified in 84% of the patients. “Dehydration”, evaluated by clinical manifestations including persistence of the skinfold, dryness of the mucosal membranes, and the presence of hypotonic ocular globus, was universal. Coma was present in only 8% of the patients. Higher values of the *[Glu]_S_*, *[Na]_S_*, serum osmolarity, and serum urea were associated with higher levels of dehydration; high values of the serum osmolarity, serum urea, and *[Na]_S_*, plus low plasma pH values, were associated with coma. The mortality rate was 16.9%, with 31% of the deaths caused by septic shock.

## 4. Studies Assessing Risks Created by Hypernatremia in Hyperglycemic Syndromes

Fulop and co-authors [29] analyzed the biochemical parameters associated with coma in 85 DKA and 47 HHS episodes. In the DKA group, both the *[Glu]_S_* and serum osmolarity were statistically higher in patients with deep coma than in alert patients, while the *[Na]_S_* was numerically, but not statistically, higher in patients with coma (136.4 ± 3.1 mmol/L) than in alert subjects (131.6 ± 0.8 mmol/L), and the arterial pH values did not differ statistically. In the HHS group, the *[Glu]_S_* (deep coma patients 51.4 ± 5.4 mmol/L, or 926 ± 97 mg/dL, and alert patients 29.7 ± 1.8 mmol/L, or 535 ± 32 mg/dL), *[Na]_S_* (deep coma patients 145.9 ± 2.6 mmol/L and alert patients 131.8 ± 0.9 mmol/L), and serum osmolarity minus serum urea concentration were statistically higher in patients with deep coma, while the arterial blood pH did not differ between the groups. The role of high *[Na]_S_* values in the development of coma in hyperglycemia has been stressed in other reports [30,31]. However, the degree of acidosis was also found to be a key contributor to hyperglycemic coma in other studies [32,33].

Wachtel and co-authors [34] investigated factors associated with in-hospital mortality related to HHS in 111 patients. Among these patients, 23 (17%) died during admission. The *[Glu]_S_* did not differ between patients (59.4 ± 8.3 mmol/L, or 1079 ± 330 mg/dL) and survivors (60.7 ± 18.2 mmol/l, or 1092 ± 328 mg/dL). The deceased patients had a significantly higher *[Na]*_S_ (148.0 ± 15.7 mmol/L) than the survivors (137.4 ± 12.2 mmol/L). In addition, the deceased patients had a higher age, serum osmolarity, and blood urea nitrogen and a higher percentage lived in nursing homes prior to being admitted with HHS.

Singhal and collaborators [35] compared biochemical parameters in two groups of diabetic patients with hyperglycemia, 41 patients with rhabdomyolysis (group 1) and 36 patients without rhabdomyolysis (group 2). The *[Na]_S_* was 148.8 ± 2.1 mmol/L in group 1 and 135.0 ± 1.1 mmol/L in group 2. The corresponding *[Glu]_S_* levels were 35.6 ± 4.5 mmol/L (640.8 ± 80.3 mg/dL) in group 1 and 24.2 ± 3.2 mmol/L (436.0 ± 56.7 mg/dL) in group 2. The differences between the groups were statistically significant for both the *[Glu]_S_* and *[Na]_S_*. Hypernatremia was present in 24 patients in group 1 (58.5%) and only in one patient (2.8%) in group 2. The association between rhabdomyolysis and hypernatremia in hyperglycemic syndromes was also detected in several case reports (vide infra). The combination of hyperglycemia and hypernatremia was also identified as a risk factor for hypogonadism in post-menopausal women [36] and for intraventricular brain hemorrhage in preterm infants [37].

## 5. Case Reports of Hypernatremia in Hyperglycemic Syndromes

The case reports included in this section consisted of 158 cases [26,27,38,39,40,41,42,43,44,45,46,47,48,49,50,51,52,53,54,55,56,57,58,59,60,61,62,63,64,65,66,67,68,69,70,71,72,73,74,75,76,77,78,79,80,81,82,83,84,85,86,87,88,89,90,91,92,93,94,95,96,97,98,99,100,101,102,103,104,105,106,107,108,109,110,111,112,113,114,115,116,117,118,119,120,121,122]. Several reports presented more than one case. The information missing in a few reports included patient age, gender, acid–base status, renal function, and outcomes of the hyperglycemic syndromes. One report [61] provided an elevated *[Glu]_S_* value and stated that hypernatremia was present but did not provide a *[Na]_S_* value, while two other reports provided *[Na]_S_* values in the range of hypernatremia and stated that the *[Glu]_S_* was higher than 55.6 mmol/L (1000 mg/dL) [93] or 27.8 mmol/L (500 mg/dL) [108] without providing the actual *[Glu]_S_* values. These three reports were included in the analysis of clinical features. The remaining 155 case reports provided the actual *[Glu]*_S_ and *[Na]_S_* values. Table 2 shows the *[Glu]_S_*, *[Na]_S_*, serum tonicity, and *[Na]_Cor_* values in the 155 cases. The table provides the medians and interquartile ranges because the Shapiro–Wilk test showed that the distribution of the values was not normal. 

The guidelines for the definition of HHS include a serum tonicity > 320 mOsm/L [11,123]. Serum tonicity values ≥ 320 mOsm/L are associated with a high incidence of lethargy and coma in hyperglycemic syndromes [124]. The serum tonicity exceeded 320 mOsm/L in all but 1 (0.6%) of the 155 case reports in Table 2, with 33 values (21.3%) ≥ 400 mOsm/L. A *[Na]_S_* ≥ 160 mmol/L indicates severe dehydration in hypernatremia without hyperglycemia [125]. A *[Na]_Cor_* ≥ 160 mmol/L also signifies severe dehydration when the *[Na]_Cor_* accurately reflects the change in the *[Na]_S_* with the correction of hyperglycemia and no change in the body water, sodium, and potassium. Among the 155 cases in this section, 12 (7.7%) had a *[Na]_Cor_* < 160 mmol/L, with the lowest value 151.2 mmol/L [86], and 5 cases (3.2%) had a *[Na]_Cor_* > 200 mmol/L, with the highest value 238.6 mmol/L [64]. The guidelines for HHS include a *[Glu]_S_* ≥ 33.3 mmol/L (600 mg/dL) [11]. This section contains 27 cases (17.4%) with *[Glu]_S_* values < 33.3 mmol/L, with the lowest value 20.3 mmol/L [69], because the aim of this review was to analyze hypernatremia in all hyperglycemic emergencies, not only in HHS. 

Hypernatremia may be encountered in hyperglycemia at all ages. In this section, the reported age of the patients at the hyperglycemic episode ranged between 5 days [59] and 87 years [117]. Infants, children, and adolescents up to 18 years of age accounted for 44 case reports [59,64,65,67,69,75,86,97,98,99,100,101,103,106,108,118,120,121]. The gender of patients, 68 females and 82 males, was provided in 150 case reports. Many patients, particularly infants and children, had no previous history of diabetes. Most of the patients had HHS, but several patients, particularly infants or children, had DKA-HHS [27,40,47,49,51,59,64,67,93,99,101,103,106,108,112,115,117,118,120,121].

The clinical features of the case reports did not differ from those reported in studies of HHS or DKA. Certain features are worth analyzing. In the study of HHS by Piniés and co-investigators [28], infections were the most common precipitating conditions of HHS, with respiratory and urinary tract infections the number 1 and 2 causes. Infections were also, by far, the most common precipitating conditions in the case reports included in this review. Fever and leukocytosis were reported in most patients on admission. Infections listed in the case reports included pneumonia [48,51,56,58,61,70,71,72,74,82,88,97,113,116,117], urinary tract infection [62,70,74,104,108,119], bacteremia [27,69,100], pulmonary tuberculosis [40], acute parotitis [47], suppurative pancreatitis [50], infected varicose veins [60], decubitus infection [70], and gastroenteritis in children [75].

Other reported conditions precipitating episodes of DKA or HHS with hypernatremia included pancreatitis [27,52,61,83], corticosteroid excess, either after administration as treatment [57,70,112], or in Cushing’s syndrome [109], heat stroke [65], diarrhea in infants [65,69], pancreatic carcinoma [56,80], discontinuation of diabetic medications [58,70,102], intravenous hyperalimentation [73], excessive intake of food items containing glucose and electrolytes [64,100,101,106,117,118,121], and extensive burns [38,42,55,66,86]. The two last conditions merit comment. An excessive intake of glucose and electrolytes, reported mainly in children, decreases the magnitude of the fluid and electrolyte deficits present in severe hyperglycemic syndromes and should be accounted for in calculating the volume of the replacement solutions. In burns, the development of hyperglycemia in non-diabetic patients has been attributed to high blood levels of endogenous corticosteroids, an excessive and very frequent intake of glucose [66], and probably transient acute pancreatitis [55].

Several patients developed hyperglycemic emergencies after days or even weeks in the hospital after admission for another medical condition. Among those admitted for the hyperglycemic emergency, the great majority had a history of polydipsia and polyuria for days or weeks. Infants were the exception. Severe weight loss was reported in many cases. Coma precipitated the admission to the hospital in several instances. Less frequently, patients had abdominal pain and vomiting. Few patients were admitted with cerebrovascular events, or falls with head injuries, or developed hyperglycemia post-operatively after brain surgery. An almost universal finding on admission was “dehydration” detected by physical examination and in some cases by invasive vascular procedures (e.g., central venous pressure measurement). “Dehydration” was associated with abnormally high levels of serum urea and creatinine in most cases. The term “dehydration” addresses only one component of the body fluid deficits in hyperglycemia.

The body fluid deficits in severe hyperglycemic episodes include dehydration and hypovolemia. The distinction between the two deficits has been stressed in several publications [126,127,128]. Bhave and Neilson specifically addressed the combination of dehydration and hypovolemia in hyperglycemic episodes [129]. The term dehydration indicates a relative water deficit in both the intracellular and the extracellular compartment and an excess of effective body solute over body water [126] and is detected by abnormally high values of serum tonicity indices [130]. Dehydration may be encountered in the presence of low, normal, or increased effective body solutes [125] and is recognized in hyperglycemia by values of *[Na]_Cor_* above the normal range of euglycemic *[Na]_S_* [6]. Hypovolemia indicates an extracellular volume deficit secondary to the loss of sodium salts and fluid [128]. In hyperglycemia, hypovolemia results from osmotic diuresis and, in some cases, from fluid losses from other sites (gastrointestinal system and skin) and causes decreased effective arterial volume and malfunction of organs, frequently of the kidneys. The majority of the 158 patients in this section had both dehydration and hypovolemia. Several case studies reported large fluid and/or weight deficits at presentation and treatment with an infusion of large volumes of hypotonic solutions containing sodium and potassium salts. 

The outcomes of 154 patients were reported. Among these patients, 50 died. The hyperglycemic episodes apparently worsened the clinical course and probably contributed to the death of 6 patients with burns [38,42,86] and 15 patients with severe infections, malignancies, hemorrhagic pancreatitis, and hepatic cirrhosis [27,50,51,52,56,61,70,72,75,80,81,88,97]. The hyperglycemic syndromes or their complications appear to have been the cause of the remaining 29 deaths [27,49,54,56,57,58,67,71,74,77,78,79,83,87,89,93,97,98,99,105,121]. Note that a few publications reported more than one death. 

Severe complications of the hyperglycemic syndromes or of their treatment included rhabdomyolysis in 13 patients [84,85,87,89,90,91,92,93,95,110,116,118,121]; osmotic myelinolysis in 11 patients [51,61,81,83,86,88,104,117]; arterial and/or venous clotting in vital organs or with adverse outcomes, e.g., leg or bowel gangrene, in 8 patients [27,77,78,100,103,108,119]; renal failure not completely recovered in 4 patients [38,50,77,87] and requiring short term dialysis by various methods in another 4 patients [89,90,98]; and one each with cerebral edema [73], pulmonary edema after saline infusion [82], profound hypokalemia (1.8 mmol/L) leading to fatal cardiac arrest [97], and parenchymal hemorrhages [98]. Again, several reports contained more than one case with the same complication. 

## 6. Fractional Losses of Body Water and Monovalent Cations in Hyperglycemia

Fluid deficits secondary to hyperglycemia cause severe clinical manifestations and treatment difficulties in patients with preserved renal function. There are issues with the accuracy of the estimated fluid loss of these patients. In contrast, the management of fluid balance in hyperglycemic episodes in patients with oligo-anuria is simple. These patients usually present with no fluid loss, may exhibit modest or no clinical manifestations [131,132,133], and are usually treated with insulin only and with monitoring of the clinical picture and the relevant biochemical indicators [134].

Difficulties in estimating accurately the fluid deficits by clinical methods are encountered in hypovolemic patients in general [128]. Specifically in hyperglycemia, large differences were reported between estimates of body fluid loss by physicians at presentation of children with DKA and retrospective calculations by nurses who used the gain in weight during treatment for their estimates [135]. The replacement fluids in hyperglycemic patients with preserved renal function must quantitatively replete the deficits of water, sodium, and potassium. Regarding the deficit of water, Adrogué and Madias in their review of hypernatremia [136] stated that the conventional formula to estimate water deficits in hypernatremia, water deficit = total body water × (1 − 140/*[Na]_S_*), with the total body water calculated as 0.6 × body weight originally [137], underestimates the water deficit in patients with losses of hypotonic fluids containing sodium and potassium. Indeed, a much larger volume of half-normal saline than water is required to correct the same hypernatremic *[Na]_S_* value [125].

This section addresses the computation of the fractional losses in the body water and monovalent cations in hyperglycemia. It includes calculations of both the deficit from dehydration and the deficit from hypovolemia and potassium loss. The calculation of these losses is based on the levels of the *[Glu]_S_* and *[Na]_S_* and the sum of the concentrations of sodium plus potassium in the fluids lost. Using these three parameters, we developed formulas computing the fractional losses of water and monovalent cations that will result in any given combination of the *[Glu]_S_* and *[Na]_S_*. Table 3 presents the abbreviations used for these formulas. In the abbreviations, subscript 1 denotes the euglycemic baseline state, and subscript 2 denotes the state of hyperglycemia. Although this report focuses on hyperglycemia with hypernatremia, these formulas apply to a large range of combinations between the *[Glu]_S_* and *[Na]_S_*.

Table 4 contains the formulas expressing the fractional losses of the water and monovalent cations in hyperglycemia.

The key formulas compute the fractional losses of the body water (Formula (9)) and monovalent cations (Formula (10)).

The current understanding of the determinants of the *[Na]_S_* in euglycemia has been based on the pivotal work of Edelman and co-investigators [138]. These authors derived a multiple linear regression formula, Formula (1) in Table 4, expressing the sodium concentration in serum water (*[Na]_SW_*) as a function of the total exchangeable body sodium, total exchangeable body potassium, and total body water. The formulas in Table 4 expressing the fractional deficits of the body water and monovalent cations are based on Formula (2), the Rose formula [139]. This formula represents a simplified expression of the determinants of the *[Na]_S_* based on the Edelman formula. Most formulas addressing the management of dysnatremias are based on the Rose formula [140].

Formula (3) expresses the sum of the body monovalent cations determining the *[Na]_S_* in the baseline euglycemic state, i.e., in the absence of excess solute with an extracellular distribution other than sodium salts. This formula was derived by rearranging Formula (2). Formulas (2) and (3) are not appropriate for estimating the water deficits in hypertonic states caused by hyperglycemia or the gain of any solute with an extracellular distribution other than a sodium salt [2,3,125]. In hyperglycemia, the *[Na]_S_*_2_ underestimates the degree of the water deficit because the *[Na]_S_* rises during the correction of the hyperglycemia without any change in the body water or electrolytes. The serum tonicity formula overestimates the degree of the water deficit because the tonicity decreases during the correction of hyperglycemia with no change in the body water or electrolytes [7]. In addition, water deficits differ between hyperglycemic syndromes with the same tonicity values but differing *[Glu]_S_*_2_ and *[Na]_S_*_2_ values, as is shown subsequently. 

The parameter that should be used for the computation of the water deficit in hyperglycemia is *[Na]_Cor_* [3,6,14,125,141,142,143,144,145]. At very high serum tonicity levels with different *[Glu]_S_* and *[Na]_S_* levels, the differences in the degree of dehydration indicated by *[Na]_Cor_* are great. For example, in the case report of Soni and coauthors [146], the *[Glu]_S_*_2_ was 311.3 mmol/L (5603.4 mg/dL) and the *[Na]_S_*_2_ was 105 mmol/l, for a serum tonicity [23] of 2 × 105 + 311.3 = 528.3 mmol/L and a *[Na]_Cor_* by the Al-Kudsi formula [24], Formula (4) in Table 4, of 105 + 1.6 × (311.3 − 5.6)/5.6 = 193.1 mmol/L. If another episode had the same tonicity of 528.3 mmol/L but a *[Glu]_S_*_2_ of 55.6 mmol/L (1000 mg/dL), the *[Na]_S_*_2_ would be (528.3 − 55.6)/2 = 238.4 mmol/L and the *[Na]_Cor_* by the Al-Kudsi formula, which is appropriate for this level of *[Glu]_S_* [3,6], would be 238.4 + 1.6 × (55.6 − 5.6)/5.6 = 250.8 mmol/L. Note that the Al-Kudsi formula, which uses Katz’s coefficient of a change in the *[Na]_S_* by 1.6 mmol/L in the opposite direction of each change in the *[Glu]_S_* by 5.6 mmol/L [147], may compute inappropriately high values of the *[Na]_Cor_* in cases of extreme hyperglycemia. Calculations of progressively smaller coefficients of a decrease in the *[Na]_S_* at progressively higher *[Glu]_S_* values have been reported [3,148,149,150] but have not been documented by actual patient data. 

Formula (5) expresses the body monovalent cations and *TBW* determining the *[Na]_Cor_* according to Formula (3). Note that *TBW*_2_ = *TBW*_1_ − *V_Lost_*, where *V_Lost_* is the volume of water lost during the development of hyperglycemia. Formula (6) computes the sum of the monovalent cations in the body fluids lost during the development of hyperglycemia. Formula (7), which was derived from Formulas (5) and (6), expresses the monovalent cation conservation during the development of hyperglycemia. Formula (8), developed by solving Formula (7) for *V_Lost_*, expresses *V_Lost_* as a function of the *TBW*_1_ and the relevant solute concentrations. Formula (9) expresses the fractional loss of body water, computed by dividing *V_Lost_* (Formula (**8**)) by *TBW*_1_. Note that at *[Na]_Lost_* + *[K]_Lost_* = 0, this formula is the same as the formula for calculating the water deficit in the Adrogué and Madias report [136] and provides the smallest possible estimate of the fractional water deficit applicable only when there is no monovalent cation deficit at any elevated *[Na]_Cor_* value. Formula (9) is a key element of Formula (10), which computes the fractional loss of the monovalent cations by dividing the sum derived from Formula (6) by *[Na]_S_*_1_ *× TBW*_1_ using Formula (8) to calculate *V_Lost_*. 

We present examples of the application of Formulas (9) and (10) under the following assumptions: (a) *[Na]_S_*_1_ > *[Na]_Lost_* + *[K]_Lost_*. Under this assumption, *[Na]_Cor_* > *[Na]_S_*_1_. (b) *[Na]_S_*_1_ = 140 mmol/L. (c) All the losses of the water and monovalent cations were through osmotic diuresis. We calculated the fractional losses in hypothetical and reported sets of hyperglycemic syndromes. For these calculations, three values of the sum *[Na]_Lost_* + *[K]_Lost_* were used: 0, 60, and 110 mmol/L. The sums of 60 and 115 mmol/L represent the lowest [20,151] and highest [18] mean urine values computed from the published studies [3]. We used 110 mmol/L in our examples because the value of 115 mmol/L contains the urine concentration of magnesium in addition to sodium and potassium [18]. 

An example is provided for *[Na]_S_*_2_ = 145 mmol/L, *[Glu]_S_*_2_ = 55.6 mmol/L (1000 mg/dL), and *[Na]_Cor_* = 145 mmol/L + 1.6 × (55.6 mmol/L − 5.6 mmol/L)/5.6 mmol/L = 159.4 mmol/L. (a) *[Na]_Lost_* + *[K]_Lost_* = 0. The body water fractional loss: (159.4 – 140)/140 = 0.139. (b) *[Na]_Lost_* + *[K]_Lost_* = 60 mmol/L. The body water fractional loss: (159.4 − 140)/(159.4 − 60) = 0.195. The monovalent cation fractional loss: 0.195 × 60/140 = 0.084. (c) *[Na]_Lost_* + *[K]_Lost_* = 110 mmol/L. The body water fractional loss: (159.4 − 140)/(159.4 − 110) = 0.393. The monovalent cation fractional loss: 0.393 × 110/140 = 0.309.

**Figure 1:**
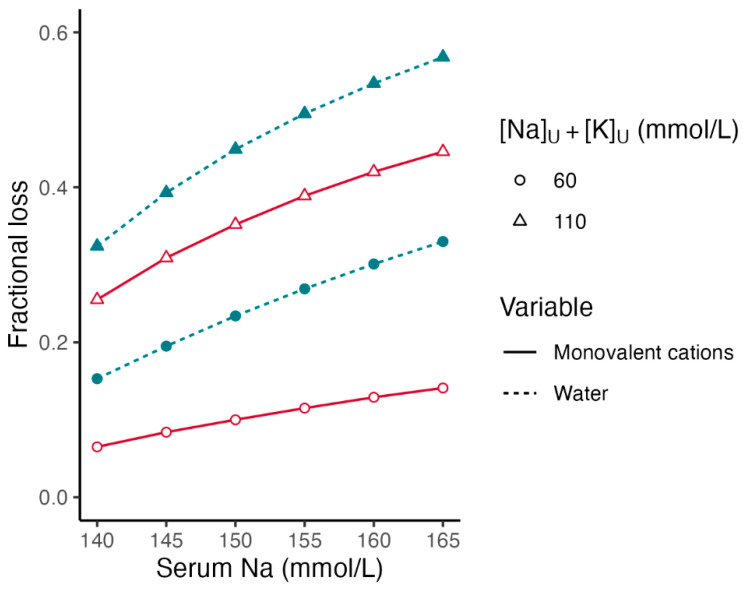
Fractional losses of body water and monovalent cations in hyperglycemia. Effect of presenting serum sodium concentration and monovalent cation concentration in the fluids lost. Legend. Fractional losses were calculated by Formulas (9) and (10) (Table 4). Serum glucose is 55.6 mmol/L (1000 mg/dL) in all calculations. Serum sodium at hyperglycemia ranges between 140 and 165 mmol/L. Red color lines: [Na]_U_ + [K]_U_ = 60 mmol/L. Blue color lines: [Na]_U_ + [K]_U_ = 110 mmol/L.

Figure 1 shows the fractional losses of the *TBW* and monovalent cations with the same *[Glu]_S_*_2_ (55.6 mmol/L), a *[Na]_S_*_2_ between 140 and 165 mmol/L, and *[Na]_Lost_* + *[K]_Lost_* at 60 and 110 mmol/L. 

**Figure 2:**
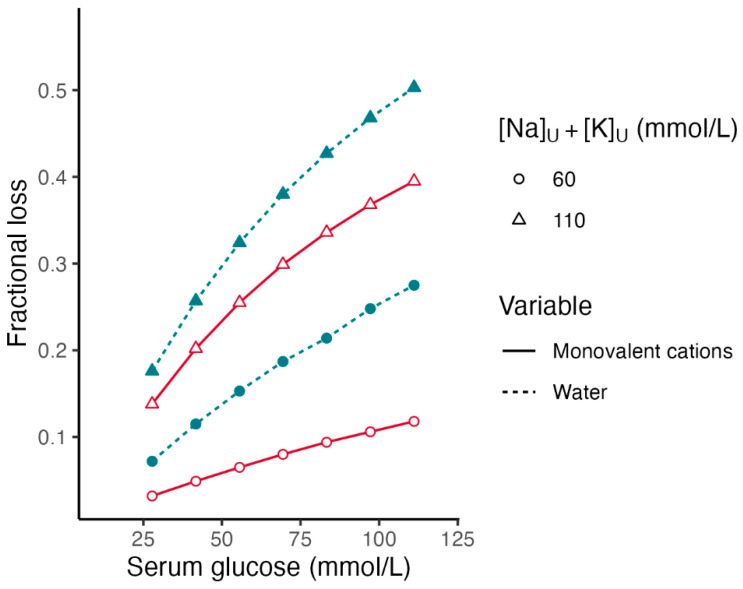
Fractional losses of body water in hyperglycemia. Effect of presenting serum glucose concentration and monovalent cation concentrations in the fluid lost. Legend. Fractional losses were computed using Formulas (9) and (10). Serum sodium in hyperglycemia is 140 mmol/L in all calculations. Serum glucose ranges between 27.8 mmol/L (500 mg/dL) and 111.1 mmol/L (2000 mg/dL). Red color lines: [Na]_U_ + [K]_U_ = 60 mmol/L. Blue color lines: [Na]_U_ + [K]_U_ = 110 mmol/L.

Figure 2 shows the fractional losses of the *TBW* and monovalent cations with the same *[Na]_S_*_2_ (140 mmol/L), a *[Glu]_S_*_2_ between 27.8 and 111.1 mmol/L (500 and 2000 mg/dL), and the two sums of *[Na]_Lost_* + *[K]_Lost_*. 

Figure 1 and Figure 2 show that in addition to the *[Na]_S_*_2_ and *[Glu]_S_*_2_, the sum *[Na]_Lost_* + *[K]_Lost_* has significant effects on the fractional losses of the water and monovalent cations in hyperglycemic syndromes. For the same *[Glu]_S_*_2_ and *[Na]_S_*_2_, the higher the sum *[Na]_Lost_* + *[K]_Lost_*, the greater the fractional losses of water and monovalent cations.

Table 5 shows the fractional losses of the body water and monovalent cations using the average values for the *[Glu]_S_*_2_, *[Na]_S_*_2_, and *[Na]_Cor_* in DKA and HHS computed in a review [6]. 

Three sums of *[Na]_Lost_* + *[K]_Lost_*, 0, 60 and 110 mmol/L, were used in Table 5. The mean *[Na]_Cor_* value in 7812 DKA cases was in the range of normonatremia, suggesting similar fractional losses of body water and monovalent cations. In this case, hypovolemia represents the only fluid deficit and should be replaced by isotonic solutions. Hypotonic solutions will only be needed later if the *[Na]_Cor_* rises during treatment, secondary to osmotic diuresis caused by high *[Glu]_S_* levels [143]. The mean *[Na]_Cor_* in 755 HHS cases was in the range of severe hypernatremia, suggesting a substantially higher loss of body water than of monovalent cations. In this case, isotonic solutions may be first infused to correct symptomatic hypovolemia, but there is clearly a need for hypotonic fluid replacement overall. 

Table 6 provides the fractional losses of the water and monovalent cations for the median *[Na]_Cor_* and values of the *[Na]_Cor_* in the interquartile range in the 155 cases with hyperglycemia and hypernatremia in Table 2 and the same *[Na]_Lost_* + *[K]_Lost_* sums as Table 5. 

Table 5 and Table 6 illustrate the differences in the fractional losses through *V_Lost_* between the *[Na]_Cor_* values in the normonatremic and hypernatremic ranges, the effects of the sum *[Na]_Lost_* + *[K]_Lost_* shown in the hypothetical examples, and the fact that the lowest fractional loss of body water is observed at *[Na]_Lost_* + *[K]_Lost_* = 0.

The key finding of this section is that the fractional losses of water, sodium, and potassium can vary widely in hyperglycemic episodes presenting with the same *[Glu]_S_*_2_ and *[Na]_S_*_2_ values and this variation is due, in large part, to different sums of *[Na]_Lost_* + *[K]_Lost_*. In two hyperglycemic episodes with the same baseline euglycemic body sodium, potassium, and water, and the same level of *[Glu]_S_*_2_ and *V_Lost_* but different sums of *[Na]_Lost_* + *[K]_Lost_*, the episode with the higher *[Na]_Lost_* + *[K]_Lost_* will have lower values of *[Na]_S_*_2_ and *[Na]_Cor_* because of a lower sum of body sodium plus potassium. A larger *V_Lost_* would be required for this episode to have the same values of the *[Na]_S_*_2_ and *[Na]_Cor_* as the episode with the lower sum of *[Na]_Lost_* + *[K]_Lost_*. This suggests that the sum *[Na]_Lost_* + *[K]_Lost_* should be considered in the calculation of the volume and composition of replacement solutions in hyperglycemic syndromes, along with *[Na]_Cor_*. The selection of the volume and composition of the replacement solutions is further complicated by the fact that the sum *[Na]_Lost_* + *[K]_Lost_* is usually not known with certainty and by the fact that the *[Na]_Cor_* may be affected by the ingestion of food items containing glucose and monovalent cations in addition to losses. For example, hyperglycemic episodes with both the *[Na]_S_*_2_ and *[Na]_Cor_* values exceeding 200 mmol/L were observed in two infants fed with inappropriately diluted formula [64].

The following sequence of actions for the use of Formulas (9) and (10) is proposed. During history taking, detailed inquiries should address urinary and gastrointestinal losses and the intake of sugar and salt by mouth or injection. Physical examination, assisted by specific tests, such as measuring the diameter of the inferior vena cava or both the body water and extracellular volume by bioimpedance [152], should address the presence and severity of hypovolemia. The advantages of bioimpedance include ease of application, patient comfort and safety, and the ability to repeat the measurements frequently. However, this method, which has been applied extensively in evaluating the fluid status of hemodialysis patients, has been found to be inaccurate in comparison to standard radioisotopic dilution methods in this patient group [128]. Research on the application of bioimpedance in hyperglycemic syndromes and on the development of accurate methods of measuring the body water and extracellular volume in clinical practice is needed.

When the *[Na]_Cor_* is in the range of hypernatremia, the calculation using Formula (9) with *[Na]_Lost_* + *[K]_Lost_* = 0 should be performed in order to obtain, in conjunction with an estimate of the body water, a measure of the volume of water necessary for the correction of hypertonicity in the body water and monovalent cation amounts in the body at presentation with hyperglycemia. The deficits of body water are particularly large when hypernatremia is accompanying hyperglycemic syndromes (Table 6). Patients with hyperglycemia and hypernatremia exhibit serious clinical manifestations and mortality (Section 5). Determining whether hypernatremia increases the frequency of adverse outcomes, which are encountered also in hyperglycemic syndromes without hyponatremia, would require studies, not case reports.

When hypovolemia is also diagnosed, the fractional deficits of the body water and monovalent cations calculated by Formulas (9) and (10) using initially a relatively low *[Na]_Lost_* + *[K]_Lost_* sum should guide the volume and composition of the infused solutions. We suggest that a *[Na]_Lost_* + *[K]_Lost_* sum of 60 mmol/L should be used in the first step of the treatment. During treatment, changes in the rate of infusion and the composition of the infused solution should be directed by the monitoring of the clinical examination and pertinent blood and urine chemistries and the urine volume [10,14,15,17,143]. The net gain in water and monovalent cations should be computed in each step. 

## 7. Limitations of the Method for Calculating Fractional Losses

The following limitations of the method for calculating fractional losses should be noted: 

(a) The deficit of sodium plus potassium in patients presenting with known body water (weight) loss over the period of development of the hyperglycemic syndrome, which may be as long as 3 weeks [151,153], may be calculated with acceptable accuracy if a reasonable estimate of body water at baseline euglycemia can be obtained. However, this calculation encounters great obstacles in patients in whom there is no information about weight loss encounters great obstacles. In these patients, monitoring of the clinical status and of the urinary losses during treatment and repeated calculations of the volume and composition of the replacement solutions based on the relevant serum tests and on the urinary losses could be the key to the application of the formulas of the present report. The clinical outcomes of these measures should be the target of research studies in hyperglycemic episodes with both known and unknown weight loss.

(b) The method does not address abnormalities of the serum potassium concentration (*[K]_S_*). Patients with preserved renal function developing hyperglycemia exhibit potassium losses through osmotic diuresis. The *[K]_S_* at presentation may vary between hypokalemia and severe hyperkalemia. During treatment, the *[K]_S_* decreases through both potassium loss in the urine from osmotic diuresis and potassium shifts into the intracellular compartment resulting from high insulin levels, decreases in tonicity, and the correction of acid–base abnormalities in some instances [154,155]. The *[K]_S_* should be monitored during treatment and its level should guide potassium salt replacement. The potassium concentration in the infused solutions should be entered as *[K]_Lost_* in Formulas (9) and (10) and the sodium concentration in these solutions should be adjusted so that the sum of the monovalent cation concentrations in the infused solutions is equal to the estimated sum of *[Na]_Lost_* plus *[K]_Lost_*.

(c) The calculation of the *[Na]_Cor_* using the Al-Kudsi formula is most probably not accurate in the early stages of the replacement of water and monovalent cations losses. The coefficients of change in the *[Na]_S_* between 1.35 and 4.0 mmol/L per the 5.6 mmol/L change in the *[Glu]_S_* have been reported in the literature [142]. Extracellular volume disorders are the leading causes of coefficients differing from Katz’s coefficient. Hypovolemia is associated with higher coefficients and hypervolemia with lower coefficients [3,6,149,156]. The effect of hypervolemia has been documented in a few anuric patients with severe hyperglycemia [157]. As noted earlier, extreme hyperglycemia may lead to low coefficients. Katz’s coefficient [147] has provided proper estimates of the *[Na]_Cor_* in most of the episodes of anuric hyperglycemia [157,158]. At presentation with hyperglycemia, patients with preserved renal function may exhibit losses of extracellular volume far exceeding the osmotic losses of intracellular volume from hypertonicity. The volume disturbances in this stage should result in increases in the *[Na]_S_* secondary to hyperglycemia smaller than those calculated using the Al-Kudsi formula [3]. The volume deficits will be corrected during treatment leading to changes in the *[Na]_S_* as the hyperglycemia is corrected progressively closer to the changes predicted by Katz’s coefficient. The *[Na]*_Cor_ should be computed after each measurement of the *[Glu]_S_* and *[Na]_S_* during treatment and should guide changes in the composition of infusion solutions [143]. 

(d) The formulas used in this method do not account for potential interactions between the osmotically active and inactive body stores of sodium and potassium, which can occur when the *[Na]_S_* changes relatively rapidly [159]. This topic needs further research. 

(e) The use of Formulas (9) and (10), as proposed in this report, has not been applied in clinical practice. Severe hyperglycemic episodes are best treated in intensive care units by intensivists applying a computer protocol containing the management procedures. Formulas (9) and (10) could be incorporated in this protocol. 

## 8. Conclusions

Hypernatremia in hyperglycemic syndromes is a sign of profound dehydration and is associated with severe clinical manifestations and mortality. In addition, hypovolemia of varying degrees is also frequent. The fractional losses of body water, sodium leading to hypovolemia, and potassium, which are routinely encountered, may vary greatly between hyperglycemic syndromes with the same values of the *[Glu]_S_*_2_, *[Na]_S_*_2_, and *[Na]_Cor_*. The main mechanisms for this variation include the sum *[Na]_Lost_* + *[K]_Lost_* and the intake of sugar and monovalent cations during the development of the hyperglycemia. An addition to the required clinical and laboratory monitoring of the calculated fractional losses of body sodium and potassium may improve the correction of water and monovalent cation deficits. 

## Figures and Tables

**Table 1:** Mean serum glucose, sodium, tonicity, and corrected sodium in reported series of hyperglycemic syndromes with mean serum sodium in the hypernatremic range.

Biochemical Parameter	Reference [26]	Reference [27]	Reference [28]
*[Glu]_S_*, mmol/L	56.1	52.2	40.7
*[Glu]_S_*, mg/dL	1009.7	940.3	732.6
*[Na]_S_*, mmol/L	153.4	153.6	153.0
Tonicity, mOsm/L	362.9	359.4	346.7
*[Na]_Cor_*, mmol/L	168.0	167.0	163.1

**Table 2:** Serum glucose, sodium, tonicity, and corrected sodium in 155 hyperglycemic case reports with sodium in the hypernatremic range.

Biochemical Parameter	Range	Median	25th Percentile	75th Percentile
*[Glu]_S_*, mmol/L	20.3–173.3	49.7	36.1	60.9
*[Glu]_S_*, mg/dL	365.0–3120.0	894.6	649.8	1096.2
*[Na]_S_*, mmol/L	145.0–228.0	160.0	152.0	169.5
Tonicity, mOsm/L	314.0–545.3	373.2	354.9	393.0
*[Na]_Cor_*, mmol/L	151.2–238.6	173.6	166.8	182.2

**Table 3:** Abbreviations used in the formulas of this report.

Abbreviation	Variable Expressed by the Abbreviation
*TBW*	Total body water, *TBW*_1_ in baseline euglycemia, *TBW*_2_ in hyperglycemia.
*[Na]_S_*	Serum sodium concentration, *[Na]_S_*_1_ in baseline euglycemia, *[Na]_S_*_2_ in hyperglycemia
*[Na]_Cor_*	Serum sodium concentration adjusted for the degree of hyperglycemia
*[Na]_Lost_*	Average sodium concentration in fluids, mainly urine, lost during development of hyperglycemia
*[Glu]_S_*	Serum glucose concentration, *[Glu]_S_*_1_ in baseline euglycemia, *[Glu]_S_*_2_ in hyperglycemia
*V_Lost_*	Volume of water lost during development of hyperglycemia
*[K]_Lost_*	Average potassium concentration in fluids, mainly urine, lost during development of hyperglycemia
*TBNa*	Total body sodium, *TBNa*_1_ in baseline euglycemia, *TBNa*_2_ in hyperglycemia
*TBK*	Total body potassium, *TBK*_1_ in baseline euglycemia, *TBK*_2_ in hyperglycemia

**Table 4:** Formulas computing the fractional losses of water and monovalent cations in hyperglycemic crises.

Formula		Reference
$[Na{]}_{SW}=1.1\times \frac{TBNa+TBK}{TBW}-25.6$	(1)	[138]
$[Na{]}_{S}=\frac{TBNa+TBK}{TBW}$	(2)	[139]
$[Na{]}_{S1}\times TBW_{1}=TBNa_{1}+TBK_{1}$	(3)	
$[Na{]}_{Cor}=[Na{]}_{S2}+1.6\times \frac{[Glu{]}_{S2}-5.6}{5.6}$	(4)	[24]
$[Na{]}_{Cor}\times TBW_{2}=TBNa_{2}+TBK_{2}$	(5)	
$\left[Na{]}_{S1}\times TBW_{1}-[Na{]}_{Cor}\times TBW_{2}=V_{Lost}\times ([Na{]}_{Lost}+[K{]}_{Lost}\right)$	(6)	
$TBW_{1}\times [Na{]}_{S1}-V_{Lost}\times ([Na{]}_{Lost}+[K{]}_{Lost})=(TBW_{1}-V_{Lost})\times [Na{]}_{Cor}$	(7)	
$V_{Lost}=TBW_{1}\times \frac{[Na{]}_{Cor}-[Na{]}_{S1}}{\left[Na{]}_{Cor}-([Na{]}_{Lost}+[K{]}_{Lost}\right)}$	(8)	
$\frac{V_{Lost}}{TBW_{1}}=\frac{[Na{]}_{Cor}-[Na{]}_{S1}}{\left[Na{]}_{Cor}-([Na{]}_{Lost}+[K{]}_{Lost}\right)}$	(9)	
$\frac{V_{Lost}\times ([Na{]}_{Lost}+[K{]}_{Lost})}{TBW_{1}\times [Na{]}_{S1}}=\frac{\frac{[Na{]}_{Cor}-[Na{]}_{S1}}{\left[Na{]}_{Cor}-([Na{]}_{Lost}+[K{]}_{Lost}\right)}\times ([Na{]}_{Lost}+[K{]}_{Lost})}{[Na{]}_{S1}}$	(10)	

*[Na]_SW_* = sodium concentration in the water fraction of the serum.

**Table 5:** Calculated fractional deficits of body water and monovalent cations through fluid loss in average cases of DKA and HHS.

Biochemical Parameter	DKA ^1^	HHS ^2^
*[Glu]_S_*_2_, mmol/L mg/dL	31.4 566	57.4 1034
*[Na]_S_*_2_, mmol/L	133.7	145.8
*[Na]_Cor_*, mmol/L	141.1	160.8
*[Na]_Lost_* + *[K]_Lost_* = 0 *V_Lost_*/*TBW*_1_	0.008	0.129
*[Na]_Lost_* + *[K]_Lost_* = 60 mmol/L *V_Lost_*/*TBW*_1_ *V_Lost_* × (*[Na]_Lost_* + *[K]_Lost_*)/(*[Na]_S_*_1_ × *TBW*_1_)	0.014 0.008	0.206 0.088
*[Na]_Lost_* + *[K]_Lost_* = 110 mmol/L *V_Lost_*/*TBW*_1_ *V_Lost_* × (*[Na]_Lost_* + *[K]_Lost_*)/(*[Na]*_1_ × *TBW*_1_)	0.035 0.028	0.409 0.322

*[Glu]_S_*_2_, *[Na]_S_*_2_, and *[Na]_Cor_* are the average values of 7812 cases of the following: ^1^: DKA [6]; and ^2^: 755 cases of HHS [6].

**Table 6:** Calculated fractional deficits of body water and monovalent cations through fluid loss in the median and the 25th and 75th percentiles of 155 cases with hypernatremia in hyperglycemia (Table 2).

Biochemical Parameter	25th Percentile	Median	75th Percentile
*[Na]_Cor_*, mmol/L	166.8	173.6	188.2
*[Na]_Lost_* + *[K]_Lost_* = 0 *V_Lost_*/*TBW*_1_	0.161	0.194	0.232
*[Na]_Lost_* + *[K]_Lost_* = 60 mmol/L *V_Lost_*/*TBW*_1_ *V_Lost_* × (*[Na]_Lost_* + *[K]_Lost_*)/(*[Na]_S_*_1_ × *TBW*_1_)	0.251 0.108	0.296 0.128	0.345 0.148
*[Na]_Lost_* + *[K]_Lost_* = 110 mmol/L *V_Lost_*/*TBW*_1_ *V_Lost_* × (*[Na]_Lost_* + *[K]_Lost_*)/(*[Na]*_1_ × *TBW*_1_)	0.472 0.371	0.528 0.415	0.584 0.459

## Data Availability

No new data were created in this study. Data sharing is not applicable to this article.
